# Efficient Deployment of Key Nodes for Optimal Coverage of Industrial Mobile Wireless Networks

**DOI:** 10.3390/s18020545

**Published:** 2018-02-10

**Authors:** Xiaomin Li, Di Li, Zhijie Dong, Yage Hu, Chengliang Liu

**Affiliations:** 1 School of Mechanical and Automotive Engineering, South China University of Technology, Guangzhou 510640, China; xiaocao0624@163.com (X.L.); itdili@scut.edu.cn (D.L.); 2 Super Micro Computer, Inc., San Jose, CA 95131, USA; yagejhu@gmail.com; 3 School of Mechanical Engineering, Shanghai Jiao Tong University, Shanghai 200240, China; chlliu@sjtu.edu.cn

**Keywords:** industrial wireless network, clustered network, maximal clique, deployment, reliability

## Abstract

In recent years, industrial wireless networks (IWNs) have been transformed by the introduction of mobile nodes, and they now offer increased extensibility, mobility, and flexibility. Nevertheless, mobile nodes pose efficiency and reliability challenges. Efficient node deployment and management of channel interference directly affect network system performance, particularly for key node placement in clustered wireless networks. This study analyzes this system model, considering both industrial properties of wireless networks and their mobility. Then, static and mobile node coverage problems are unified and simplified to target coverage problems. We propose a novel strategy for the deployment of clustered heads in grouped industrial mobile wireless networks (IMWNs) based on the improved maximal clique model and the iterative computation of new candidate cluster head positions. The maximal cliques are obtained via a double-layer Tabu search. Each cluster head updates its new position via an improved virtual force while moving with full coverage to find the minimal inter-cluster interference. Finally, we develop a simulation environment. The simulation results, based on a performance comparison, show the efficacy of the proposed strategies and their superiority over current approaches.

## 1. Introduction

Wireless networks have attracted considerable attention due to their many advantages and industrial applications, such as industrial equipment monitoring and control order transmission. In the new Industry 4.0 architecture, or smart factory, the new manufacturing system must accomplish multi-layered and ubiquitous data communication, such as machine-to-machine (M2M), machine-to-user (M2U), and machine-to-cloud (M2C) server capability [1,2]. Recently, with the increased development of smart manufacturing, a growing number of mobility elements have been introduced into the current system. Because industrial mobile wireless networks (IMWNs) can save costs and accommodate flexible deployment, IMWNs are widely used in manufacturing systems [3,4]. Wireless networks play a key and increasing role in these systems; thus, efficient deployment of network nodes and anti-interference schemes are essential for the reliable operation of IMWNs.

With the progressing research of industrial wireless networks (IWNs), network structure design and optimization has become an area of considerable interest. Previous studies have determined that a clustered network framework has the following advantages [5,6,7]: (1) Since cluster heads are responsible for most of the communication and management tasks, a clustered network structure can reduce the communication load of other nodes in a network; thus, clustered networks are easy to centrally manage. (2) In a clustered sub-system, channel interference can be reduced by time division multiple access (TDMA). (3) A group network is beneficial to the entire network’s security and privacy because such a system can easily isolate a network virus or attack. Meanwhile, compared with traditional networks, wireless networks have the following new features. Firstly, IMWNs are adopted to gather important parameters (such as gas emission) or deliver control instruction and emergent information, so low communication latency and high communication stability are required [8,9]. Secondly, in industrial domains, wireless networks consist of equipment with limited forms of energy, such as batteries in harsh environments. If the wireless nodes consume high amounts of energy, networks face low network connectivity and lifetime [10,11]. Summary, IMWNs are different from traditional wireless networks in terms of their features and performance, such as their real-time operation, reliability, and low-energy consumption. Therefore, new manufacturing systems add challenges to IMWN key node deployment (in the form of access points, cluster heads, and group leaders, for example) and increase inter-cluster interference.

Overall, network deployment can be divided into target coverage, area coverage, and barrier coverage [12]. Previous work on network node deployment has concentrated on cases in which nodes are static and deterministic. Moreover, those research studies focused on the network connectivity, energy consumption or coverage ratio. Channel interference consists of inter-clustered, intra-clustered, and neighbor interference. Studies addressing anti-interference [13,14] have made use of time slots and MIMO (multiple-input multiple-output) technologies to reduce interference from different layers. Although these studies provide beneficial references on the topic, many deficiencies exist in the current state of research: (1) Most studies independently solve deployment or anti-interference. (2) Recent works have not considered industrial applications. In this study, we jointly develop optimal strategies for cluster head deployment and inter-cluster interference by considering the needs of industrial applications. The main contributions of the present study are summarized as follows:

(1) Using the trajectory discretization method, static and mobile node coverage is simplified into a one-dimensional target coverage problem. From the perspective of a virtual cluster’s interior, we simplify the target coverage to a maximal clique problem by developing and analyzing a mathematical model.
(2) We divide the problem into two stages. In the first stage, for full target coverage and improved real-time performance, a distributed double-layer Tabu search is employed to solve the optimal number and position of cluster heads. In the second stage, we use an improved virtual force and a virtual cluster head movement to redeploy the position of the cluster heads.

This paper is organized as follows. A brief literature review on network deployment and inter-cluster interference in wireless networks is presented in Section 2. Preliminary information and the system model are provided in Section 3. Section 4 details new methods for optimal coverage via two stages. Simulations, results, and analysis are presented in Section 5. Finally, the paper concludes with a summary in Section 6.

## 2. Related Works

In this section, previous works on network deployment and wireless network anti-interference methods is reviewed.

Network coverage, which is the key problem of network deployment, directly affects the performance of the entire network and has thus drawn attention from a large number of researchers. Based on the description above, the coverage consists of target, area, and barrier coverage. Target coverage is the basic problem into which all other coverage problems can be transformed. In [15,16,17,18,19], the authors employed heuristic and improved methods to optimize target coverage from the perspective of maximum network lifespan, network coverage ratio, and error-tolerance rates; the feasibility of the algorithms were independently verified via simulation experiments. However, these algorithms focus only on the target coverage of nodes in a static state; they do not consider the mobile situation. Therefore, in [20,21], to reduce the moving distance and minimal energy consumption, the authors provided a mathematical model and used strategies based on Hungarian and greedy algorithms. Hamid et al. [22] proposed a method for updating the new location of a mobile node for a minimum of coverage holes based on the configuration of Voronoi polygons and the virtual force. However, this and similar studies focused on the conventional, environment-sensing, node coverage problem and did not consider the specific application or actual demand of the industrial environment [23,24].

In industrial wireless network deployment research, another report [25] reviewed and discussed the applicability and limitation of different methods for target coverage in industrial applications; the simulation results demonstrated the method’s advantages and disadvantages via different evaluation indicators. The authors of [26] jointly optimized node deployment and dynamical sleep scheduling via a hybrid harmony search and genetic algorithms. Another study [27] decisively solved the connectivity problem that occurs when industrial wireless sensors are grouped, established a wireless network and inter-group connection system model, and optimized the deployment of nodes in the industrial environment. Industrial wireless network deployment studies have focused on single performance indexes of wireless networks in industrial environments, such as energy consumption, connectivity, or moving distance. However, the key indicators of IWNs, such as real-time stability, have been given less consideration.

As wireless network research has developed, an increasing number of studies have shown that grouped or clustered network frameworks have a strong advantage over network management, security, and other features [28]. However, inter-cluster interference becomes a bottleneck problem, particularly in single-radio-frequency systems. The authors of [29] divided M2M wireless communication interference into inter-cluster and intra-cluster interference and then employed a mixed integer nonlinear programming method to manage radio resources. The authors of [30,31] introduced an adaptive time-slot dynamic, self-management mechanism for increasing the inter-cluster interference; the simulation results verified the strategy. In addition, previous studies [32,33,34] have increased the reliability of clustered networks by adopting dynamic sleep, MIMO, and cognitive radio technologies. However, these methods require high-resolution time synchronization and neighbor node information; thus, they are not easily applied in the industrial domain.

## 3. Preliminaries

### 3.1. Network Model and Hypothesis

In industrial environments, smart manufacturing systems employ hybrid networks, including both static and mobile nodes, thus increasing mobility and flexibility. To facilitate modeling of this type of problem without losing generality, the present study introduces the following network assumptions. (1) Networks adopt a single wireless frequency radio band. (2) Network nodes work with omnidirectional antennas. (3) Mobile node paths remain unchanged. Based on these assumptions, we present the following clustered IMWN model. 

Let *A* denote the industrial environment on a 2D plane. We assume that the industrial environmental interferences of environment are static, so a fixed effective communication range is used. Consider a set of *n* ≥ 1 static nodes in *A*, denoted by $S^{C}=\{s_{1}^{c},s_{2}^{c},\cdots ,s_{n1}^{c}\}$, with a communication range of $r_{i}^{c}$. Each node $s_{i}^{c}$ has a unique ID number and a coordinate location $\left(x_{i}^{c},y_{i}^{c}\right)$. The main task of the static node is to sense key parameters from the physical world and then to transmit or receive the data to or from clustered heads. Let $D^{C}=\{d_{1}^{c},d_{2}^{c},\cdots ,d_{n}^{c}\}$ be the set of communication loads of static nodes *S^c^*. A cluster is a sub-system. A finite number of cluster heads *Z* > 1 work in *A*. Let $AP=\{ap_{1},ap_{2},\cdots ,ap_{z}\}$ be the set of cluster heads. A cluster head *ap_i_* has the coordinate location $\left(x_{i}^{ap},y_{i}^{ap}\right)$, with an efficient communication range $r_{i}^{ap}$. Assume that IMWN *A* has m > 0 mobile nodes, denoted by $S^{R}=\{s_{1}^{r},s_{2}^{r},\cdots s_{m}^{r}\}$, with the communication range $r_{i}^{r}$. Let $D^{R}=\{d_{1}^{r},d_{2}^{r},\cdots ,d_{m}^{r}\}$ be the set of communication loads of static nodes *S^R^*. Meanwhile, for a better understanding of the problem, assume that *t* communication loads of wireless static and mobile nodes are fixed. Mobile nodes move along the path via vision, magnetic wires, ribbons, and so on. Let $L=\{l_{1},l_{2},\cdots ,l_{m}\}$ and $V=\{v_{1},v_{2},\cdots ,v_{m}\}$ be the set of paths and velocity, respectively.

### 3.2. Network Model and Hypothesis

**Figure 1:**
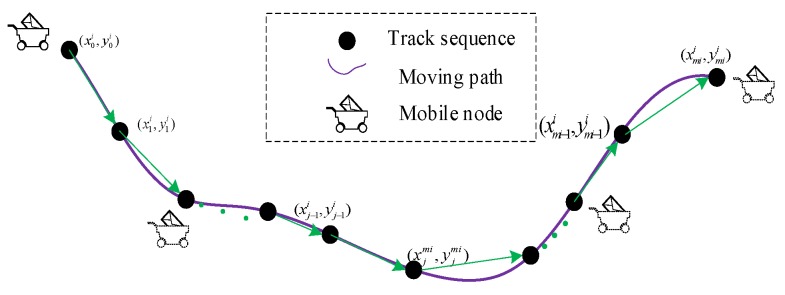
Discrete and continuous moving paths.

Discretization of the mobile node trajectory: Assume that mobile node $s_{i}^{r}$ communicates with cluster head *AP_j_* over time *t*, as shown in Figure 1. Therefore, we obtain the mobile node moving a distance *d_m_* = *v_i_* × *t* during the period, and the coordinate location of $s_{i}^{r}$ changes from $\left(x_{j-1}^{i},y_{j-1}^{i}\right)$ into $\left(x_{j}^{i},y_{j}^{i}\right)$. Thus, according to the rule, *l_i_* can be divided into *m_i_* sections. In other words, we can use a series of discrete points to represent the moving path.

**Definition** **1.** Communication trajectory sequence: The communication trajectory sequence T_i_, of mobile nodes $s_{i}^{r}$, along path l_i_ is the set that contains a series of moving positions over *t*. Namely, $T_{i}=\{(x_{0}^{i},y_{0}^{i}),(x_{1}^{i},y_{1}^{i}),\cdots ,(x_{mi}^{i},y_{mi}^{i})\}$.

According to Definition 1, we can use a discrete point set *T^′^* to express the set of paths L. Thus, the discrete point set *T^′^* is

(1) $$T^{\prime }=\overset{m}{\underset{i=1}{\cup }}T_{i}$$

where $T_{i}\neq \emptyset$. Through discretization and the use of Definition 1, the coverage of the moving trajectories is transformed into the covering of point sets *T^′^*.

**Definition** **2.** Full coverage: When all members s_i_ of S are covered by AP, then AP fully covers S. The symbol $AP\overset{⌢}{\;ϒ}\;S$ denotes full coverage.

**Theorem** **1.** The necessary condition for path L to be fully covered by cluster head AP is that point set T^′^ is fully covered by AP.

**Proof.**  If path *L* is covered by *AP*, then all points *P_all_* are covered by *AP*, that is, $AP\overset{⌢}{ϒ}P_{all}$. According to Definition 1, $T_{i}^{\prime }\subset P_{all}$; thus, point set *T^′^* is fully covered by *AP*.

Starting from Definitions 1 and 2 and Theorem 1, we note that the problem of full coverage can be changed into a point set coverage problem. Let *P*^′^ denote the target point set, such that $P^{\prime }=T^{\prime }\cup S^{c}$ and $n=n1+\Sigma_{i}^{m}\left\|T_{i}\right\|$. Similarly, let point set *AP* denote the cluster heads. We can obtain the Euclidean distance *d* between point $p_{i}\in P^{\prime }$ and cluster head $ap_{j}\in AP$ as follows:

(2) $$d(p_{i},ap_{j})=\sqrt{{\left(x_{i}^{p}-x_{j}^{ap}\right)}^{2}+{\left(y_{i}^{p}-y_{j}^{ap}\right)}^{2}}$$

We assume that $r_{i}^{c}=r_{i}^{r}=r_{i}^{ap}=R$. When *d* (*p_i_*, *ap_j_*), i.e., the distance between point *p_i_* and the cluster head *ap_j_*, is less than the effective coverage radius of cluster head *R*, then *ap_j_* covers the target point *p_i_*. Let *Cov_ij_*(*p_i_*, *ap_j_*) be the coverage function:

(3) $$Cov_{ij}(p_{i},ap_{j})=\{\begin{array}{cc} 1, & d(p_{i},ap_{j})\leq R,\\ 0, & \mathrm{other}. \end{array}$$

**Definition** **3.** *Optimal cluster coverage problem (OCCP): We seek the minimum AP to fully cover the target point. Given the target point set P^′^ in A, we want the minimum number and location of AP that fully covers P^′^, for which the following function is minimized.*

(4) $$N_{AP}=\mathrm{crad}(AP)$$

The problem described above is called the *OCCP*.

Wireless channel model: Assume that *P_t_* is the transmission power of *ap_j_*; then, the received power of the target point *p_i_* $\left(AP\overset{⌢}{ϒ}P\right)$ is described by the following:

(5) $$P_{r}=P_{t}-a(d_{0})-10\alpha \mathrm{lg}(d_{ij}/d_{0})$$

Let *B^i^* be the clustered neighbor set such that all cluster heads $b_{i}\in B^{i}$ satisfy $b_{i}\;\overset{⌢}{ϒ}\;p_{i}$, and let *ap*_0_ be the corresponding cluster head of *p_i_*. Therefore, we can obtain the inter-cluster interference power *P*_inf_intercluster_ of target point *p_i_* as follows:

(6) $$P_{\inf\_\mathrm{intercluster}}=\underset{bi\in Biap_{0}}{\Sigma }\left(P_{t}-a(d_{0})-10\alpha \mathrm{lg}(d_{ij}/d_{0})\right)$$

Then, the signal-to-noise ratio (*SNR*) of *p_i_,* in cluster *ap*_0_, is

(7) $$\mathrm{SNR}(P_{i})=P_{r}-P_{\inf\_\mathrm{intercluster}}$$

In Equations (5) and (6), *a*(*d*_0_) is the path loss of wireless signals with the distance *d*_0_; α is a path loss index such that α>2 in the industrial environment. In addition, let $\beta_{ij}$ be the correlation function of the two cluster heads *ap_i_* and *ap_j_*. The following function is described:

(8) $$\beta_{ij}=\{\begin{array}{c} 1,\;P_{i}^{AP}\cap P_{j}^{AP}\neq \emptyset \\ 0,\;P_{i}^{AP}\cap P_{j}^{AP}=\emptyset \end{array}$$

where $P_{i}^{AP}$ and $P_{j}^{AP}$ are the covered target point sets of *ap_i_* and *ap_j_*, respectively.

According to Equations (5)–(7), when *P_t_* is constant and the environment is determined, a decreasing *P*_inf_intercluster_ is the most effective way to increase *SNR*, which implies that *d_ij_* is increasing. Therefore, we use the effective distance *ol* between cluster heads to represent the inter-cluster interference power. For cluster head node *ap_i_*, there are *k* cluster heads with a signal overlapping area, and the cluster head node *ap_i_* for all effective overlap distances is

(9) $$ol_{i}=\frac{1}{2}\beta_{ik}\underset{k}{\Sigma }\left(2R-d_{ik}\right)$$

The total effective overlapping distance, mapped by the inter-cluster interference signals of the whole network, can be expressed by

(10) $$OL=\underset{i=1}{\Sigma }ol_{i}=\overset{\left\|AP\prime \right\|}{\underset{i}{\Sigma }}\overset{K}{\underset{k}{\Sigma }}\beta_{ik}(2R-d_{ik})$$

**Definition** **4.** *Best communicated cover problem (BCCP): We seek the best location of AP with the minimum inter-cluster interference. Given the target points set P^′^ in A, we want the minimum inter-cluster interference of AP that fully covers P^′^, for which the OL function is minimized.*

(11) $$\begin{array}{l} \mathrm{MIN}(OL)\\ \mathrm{Subject}\;\mathrm{to}.\;P^{\prime }\overset{⌢}{ϒ}AP \end{array}$$

### 3.3. Maximal Communication Latency and Maximal Energy Consumption

In the section, we will analyze the proposal from communication latency, energy consumption, and number of inter-cluster interference targets. 

Maximal communication latency (MCL): In a cluster, we will use the maximal communication latency to describe the communication real time in a cycle. Assume that in one cluster, there are κ target points and δ inter-cluster interference targets. We can get the MCL for the one cluster

(12) $$\tau =\frac{\Sigma_{i\in \lambda }D_{i}+\Sigma_{j\in \delta }\mathrm{Pr}ob_{j}D_{j}}{V_{c}}$$

where *V_c_* is the communication rate, and *Prob_j_* is the data recommunication probability for the *j*-th inter-cluster interference targets. Therefore, we can get the maximal communication latency by $\Gamma_{\max}=Max(\tau_{i})$ and $1\leq i\leq Num_{Ap}$. Consider the communication and channel energy consumption spent, we can obtain the maximal energy consumption (MEC), formulated as follows:

(13) $$E=\varsigma \Gamma_{C}+\xi \Gamma_{L}$$

where ς, ξ are the energy consumption coefficients of communication and wireless channel, respectively. 

## 4. Our System Model (Proposed Methods)

In this section, we present our algorithm. First, we employ a distributed double Tabu search method to solve the OCCP and to find the fully covered cluster head set *AP* and the associated coarse locations. Then, an improved virtual force and virtual motion strategy is adopted to find the best results of the BCCP based on the cluster head set *AP*.

### 4.1. Optimal Cluster Head Coverage Problem—Stage I

In industrial environments, coverage of all points is the main communication problem in industrial network data. From the view of a single cluster analysis, the relationship between points in the same cluster is the premise for solving the problem. In a cluster, any Euclidean distance *d_ij_* of two points (*v_i_*, *v_j_*) meets the condition $0\leq d_{ij}\leq 2R$. If any two points meet the condition, we call the two points connected. As a result, we obtain the following theorems.

**Theorem** **2.** If the two-point Euclidean distance ≤2R denotes that two points are collected, then the target point set V in a cluster is a subset of the maximal clique C that includes >V.

**Proof.**  For a cluster radius of *R*, the Euclidean distance between any two points $v_{i},v_{j}\in V$ is less than 2*R*. That is, $\forall \;\exists \;d_{ij}\leq 2R$ because, when *d_ij_* ≤ 2*R,* the two points are connected. In other words, any two target points are connected. According to the definition of the maximum clique, we obtain V⊆C. That is, *V* is a subset of the maximal clique *C*.

**Theorem** **3.** The OCCP is an NP-hard problem.

**Proof.**  According to Theorem 2, *V* is a subset of a maximal clique; thus, the OCCP should aim to find the maximal clique enumeration (MCE) of all target points of *P*^′^. MCE is an NP-hard problem. The OCCP is also an NP-hard problem.

According to Theorem 3, it is challenging to find an algorithm that obtains all possible maximal cliques of a graph in polynomial time; thus, an intelligent heuristic algorithm is used in this study.

The distributed double Tabu search method: In industrial domains the algorithms provide not only efficiency but simplicity and easily operation, the Tabu search method has the above advantages and is fit to small-scale situations. Therefore, for solving the OCCP and MCE, an improved Tabu search method is adopted in the section. Given a deterministic graph *G* = {*P*^′^, *E*}, where *P*^′^ is the target point set and *E* is the set of links, let graph *G* be divided into *f* regions:

(14) $$G=\overset{f}{\underset{i=1}{\cup }}G_{i}.$$

Different methods produce different effects; thus, we employ a complex network modularity *Q* to divide *G*:

(15) $$Q=\underset{i}{\Sigma }\left(e_{ii}-a_{i}^{2}\right)$$

where *e_ii_* is the fractional link between community *i* and the total link *G*; *a_i_* is the fraction of ends of edges that are attached to vertices in community *i*.

In this study, a greedy algorithm (previously used to maximize the degree of modularity [35]) is used to divide *G*. The working principle is as follows: First, each target point $p_{i}\in P^{\prime }$ is regarded as an independent module. Then, according to Equation (11), we compute the increment between the network modularity of any two modules *G_i_* and *G_j_*. Based on the greedy principle, we merge the two modules, which makes the maximum modularity increase and iterate the steps above stepwise until the number of modules is *f*. For a divided module *G_i_* $\left(G_{i}\subseteq G,i\leq f\right)$, let *N*(*G_i_*) be the neighbor vertex set of *G_i_* with $G_{i}\cap N(G_{i})=\emptyset$.

**Theorem** **4.** Given a point v(v∈C), which belongs to a maximal clique C, and the subgraph G_i_$\left(v\in G_{i}\right)$, then $G_{i}\cup N(G_{i})$ contains C.

**Proof.**  A maximal clique *C* includes a vertex set that is denoted by *C* = {*v*, *U*}; *U* is the only remaining vertex set after deleting *v* because $v\in G_{i}$ and, according to the clique, any members in *u* ∈ *U* are now linked with *v*. Thus, there are two cases: (1) *u* ∈ *G_i_* and (2) *u* ∉ *G_i_* and their neighbors *G_i_*. In other words, *u* ∈ *N*(*G_i_*). Therefore, $G_{i}\cup N(G_{i})$ contains *C*.

**Figure 2:**
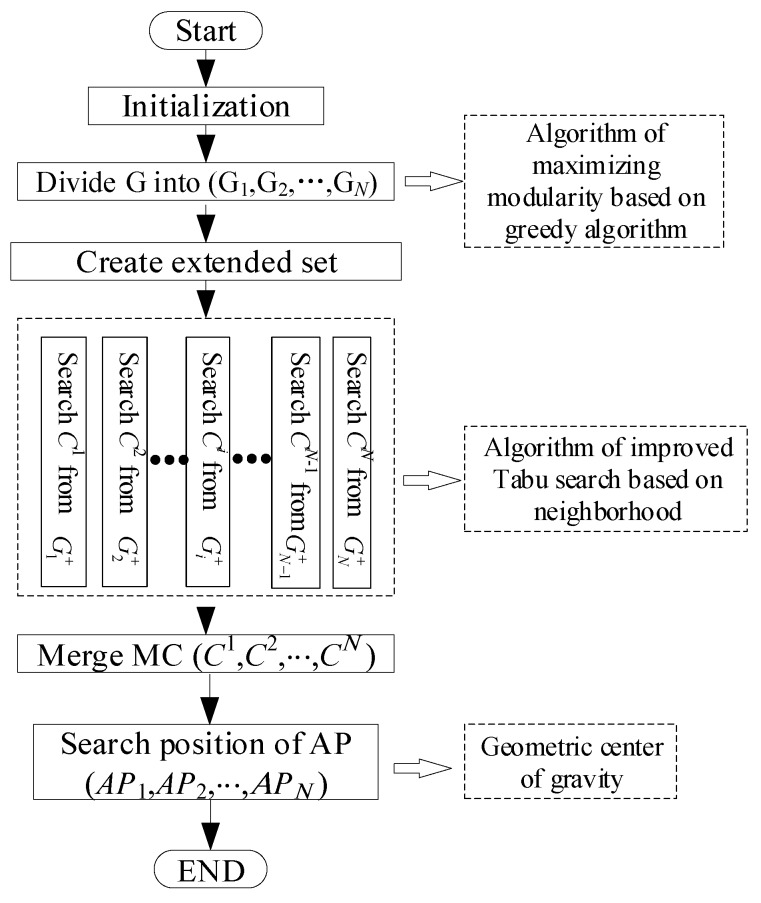
The distributed parallel improved Tabu search algorithm.

Let $G_{i}^{+}=G_{i}\cup N(G_{i})$ be the extended set of *G_i_* and $G^{+}=\{G_{1}^{+},G_{2}^{+},\cdots ,G_{f}^{+}\}$ be the extended *G*. According to the earlier discussion and the properties of the maximal clique, as shown in Figure 2, we adopt the distributed computing method to find the results. We use the double Tabu method to find the results of $G_{i}^{+}$ with the *P_i_* vertex in one subgraph, as shown in Algorithm 1. The method is explained as follows. First, we randomly select a point *p_i_*; then, we create the left vertex set of the target *P*_*left* = *P_i_*, the maximal clique set *C^i^* = {*p_i_*, *p_j_*}, *p_j_* ∈ *N*(*p_i_*), and the candidate select set *Candi*_*set* = *N*(*p_i_*) ∩ *N*(*P_j_*). In addition, the Tabu list is given such that *Tabu*_*set* = {*p_i_*, *p_j_*}. We randomly select a target pont *p*_*next* from the *Candi*_*set* to join in *C^i^* = {*p_i_*, *p_j_*, *p_next*}; then, we update *Candi*_*set* = $\underset{k\leq |C^{j}|}{\cap }N(c_{k}^{j})$ − *C^j^* until *Candi*_*set* = ø. Next, we record the *C^j^* in $C_{all}^{i}$ and delete the member *C^i^* from *P*_*left*. The vertex degree of the deleted member is less than |*Ci*| − 1. We repeat the above steps until *P_left* = ø.
**Algorithm 1:** The double-layer Tabu search maximal clique (DTSMC).
**Input**: $G_{i}^{+}$, *Degree* ($G_{i}^{+}$) and *N*($G_{i}^{+}$) **Output**: $C_{all}^{i}$
1Initialization: randomly select the min degree vertex *Current* from $G_{i}^{+}$, *Tabu_first_set* = ø, *Tabu_second_set* = ø, $C_{all}^{i}$ = ø, *C^i^* = ø, *p*_*next* = ε, *P_left* = $G_{i}^{+}$2**for**
*j* = 1; *j* ≤ |*P*_*left*|; *j*++ do // Find maximal clique of subgraph3 *Candi_set* = *N*($p_{j}^{i}$);4 **while** |*Candi_set*| ≠ ø // Find the maximum clique at the current node5  Randomly select a vertex *p_next* from *Candi_set*;6  **if**
*p_next* ∈ $\underset{k\leq |C^{j}|}{\cap }N(c_{k}^{j})$ // Evaluate common neighbor nodes7   *C^j^* ← *C^j^* ∪ {*p_next* };8   *Tabu_second_set* ← *Tabu_second_set* ∪ {*p_next*};    // Update second-level Tabu list9   *Candi_set* ← $\underset{k\leq |C^{j}|}{\cap }N(c_{k}^{j})$ − *C^j^*; //10  **end if**11 **end while**12 **for**
*k* = 1, *K* = |*C^j^*|13  **if**
*degree*($c_{k}^{j}$) = |*C^j^*| − 1 // Compute the node degree14   *P_left* ← *P_left*/$c_{k}^{j}$; // Update second-level candidate solution list15   *Tabu_firt_set* = $G_{i}^{+}$ − *P_left*; // Update first-level Tabu list16  **end if**17 **end for**18 $C_{all}^{i}$ = $C_{all}^{i}$ + *C^j^*;19**end for**20**Return**
$C_{all}^{i}$ //Return all maximal clique

Determine the cluster head number and locations: Using Algorithm 1, we can obtain the sub maximal clique set $C_{all}^{i}$ for each subgraph from $G_{i}^{+}$; then, we delete the same maximal clique, which is *C* = Merge{*C*^1^, *C*^2^, … }. Let *C =* {*c*_1_, *c*_2_, …, *c_z_*}, where each maximal clique represents a cluster head. However, to deal with communication load and obtain low communication latency, we need to determine the number of cluster heads. Consider *ki* as the number of coverage targets for *c_i_* ($c_{i}\in C$) and 1≤i≤z, in accordance with the maximum allowable communication load for a cluster head. We can compute the *i*-th cluster head number $Num_{Ap}^{i}$ and the total number of cluster heads in an entire IMWN, $Num_{Ap}$. The formulations are given by

(16) $$Num_{AP}^{i}=\left\lfloor \frac{\underset{j\in ki}{\Sigma }Dj}{D_{max}}\right\rfloor$$

(17) $$Num_{Ap}=\Sigma Num_{Ap}^{i}$$

where *D_j_* is the communication load of the *j*-th static or mobile node. *D*_max_ is the maximum allowable communication load for a cluster head, and ⌊⋅⌋ works to round up to the value. 

After the maximal cliques of target points *P*^′^ and the number of cluster heads are obtained, we determine the coarse location of each cluster head. First, we select the related number of targets for keeping commination load less than *D*_max_. We use the selected targets’ gravity center to compute the location of the cluster head. The formulations are given by

(18) $$X_{i}=\frac{\Sigma x_{j}^{Ap_{i}}}{\left|Ap_{i}\right|},\;Y_{i}=\frac{\Sigma y_{j}^{Ap_{i}}}{\left|Ap_{i}\right|}$$

where $\left(x_{j}^{Ap_{i}},y_{j}^{Ap_{i}}\right)$ denotes the coordinate location of the *j*-th target point position of *AP_i_*, and |·| obtains the number of target points in one cluster. If there are multiple cluster heads in the same maximal clique, an orthogonal frequency or MIMO technologies are adopted. 

### 4.2. Minimum Inter-Cluster Interference Problem—Stage II

From the discussion above, we know that any overlapping distances of cluster heads can express inter-cluster interference. Thus, in this section, we use an improved virtual force and virtual motion to solve the BCCP. In the traditional virtual force model, when the distance between two clusters *ap_i_* and *ap_j_* is less than 2*R*, i.e., $d_{ij}^{ap}\leq 2R$, then the virtual force is a repulsive force; otherwise, it is an attractive force. The model is not fitted to the minimal inter-cluster interference; when there are no target points in the overlap, for example, the above model is inefficient. Therefore, we use only the repulsive force to express the virtual force, and the function of inter-cluster correlation β is introduced into the model. We use the following formulation to express the virtual force:

(19) $$\vec{f_{ij}}=\{\begin{array}{ll} \beta_{ij}\cdot k\cdot ol_{ij} & d_{ij}\leq 2R\\ 0 & d_{ij}>2R \end{array}$$

where the *d_ij_* is the distance between the *ap_i_* and *ap_j_* clusters. Then, the strength of the virtual force in the *x*- and *y*-axes can be expressed as follows:

(20) $$\begin{array}{l} f_{x}^{ij}=\left|{\vec{f}}_{ij}\right|\cos\theta_{ij}=\left|{\vec{f}}_{ij}\right|\frac{x_{i}^{AP}-x_{j}^{AP}}{d_{ij}^{AP}}\\ f_{y}^{ij}=\left|{\vec{f}}_{ij}\right|\sin\theta_{ij}=\left|{\vec{f}}_{ij}\right|\frac{y_{i}^{AP}-y_{j}^{AP}}{d_{ij}^{AP}} \end{array}$$

where $\theta_{ij}$ is the angle between the virtual force and the *x*-axis. Let $N_{i}^{AP}$ be the neighboring cluster set of cluster heads *ap_i_*; we can then obtain the total virtual force $\vec{F_{i}}$ of *ap_i_* in the industrial wireless network in *A*. We use the following equation to determine the total virtual force:

(21) $$\vec{F_{i}}=\underset{\begin{array}{l} k\leq |N_{i}^{AP}|\\ k\neq i \end{array}}{\Sigma }\vec{f_{ik}}$$

It is then straightforward to obtain the total virtual force of the entire network $\vec{F}$ as follows:

(22) $$\vec{F}=\frac{1}{2}\underset{i\leq C^{all}}{\Sigma }\vec{F_{i}}$$

According to the analysis above, we minimize the virtual force to solve the BCCP and ensure that all target points are fully covered:

(23) $$\begin{array}{l} \mathrm{Min}(\vec{F})\\ \mathrm{Subject}\;\mathrm{to}.\;P^{\prime }\overset{⌢}{ϒ}AP \end{array}$$

A minimum inter-cluster interference strategy based on improved virtual force and motion: After obtaining the virtual force, we use the virtual motion of the cluster heads to reduce the virtual force. To obtain Equation (19), certain sub-problems must be solved: (1) moving the maximal distance *d_in_* and (2) finding the stop motion condition. The normal line of the virtual force $\vec{F_{i}}$ of *ap_i_* divides the members of *ap_i_* into right and left sections: $P^{Left}$ and $P^{Right}$. The left section members (target points) restrict the moving distance by meeting $P\prime \overset{⌢}{ϒ}AP$. For any member $p_{k}\in P^{Left}$, we use the following formulation to compute the $d_{k}^{p}$ of the member of *p_k_* in the cluster head *ap_i_*:

(24) $$d_{k}^{p}=\sqrt{R^{2}-d_{PF}^{2}}-\sqrt{d_{po}^{2}-d_{PF}^{2}}$$

where *d_pf_* is the distance between the left node *p_j_* and the line of the virtual force; *d_op_* is the distance between the left node *p_j_* and the cluster head *ap_i_*. Equation (19) is used to compute the maximal distance *d_in_*:

(25) $$d_{in}=\min\{\min(d_{p}),d_{move\_step}\}$$

where *dp* is the set of $d_{k}^{p}$ for the remaining members of *ap_i_* and *d_move_step_* is the iterative moving distance. After achieving the maximum moving distance *d_in_*, we use the following method to update the new location of the cluster head ($x_{next}^{AP},\;y_{next}^{AP}$) according the current position ($x_{current}^{AP},\;y_{current}^{AP}$):

(26) $$\begin{array}{l} x_{next}^{AP}=\{\begin{array}{ll} x_{current}^{AP} & if\;\left|F\right|\leq F_{TH}\\ x_{current}^{AP}+\frac{F_{x}}{F}\cdot d_{in}\cdot e^{\frac{-1}{F}} & if\;\left|F\right|>F_{TH} \end{array}\\ y_{next}^{AP}=\{\begin{array}{ll} y_{current}^{AP} & if\;\left|F\right|\leq F_{TH}\\ y_{current}^{AP}+\frac{F_{y}}{F}\cdot d_{in}\cdot e^{\frac{-1}{F}} & if\;\left|F\right|>F_{TH} \end{array} \end{array}$$

where *F_x_* and *F_y_* are the virtual forces in the *x*- and *y*-axis, respectively, and *F_TH_* is the threshold value. 

The details of the proposal are given in Algorithm 2 (VFM). The method is explained as follows. Suppose that there are *Z* clusters and that their position is *AP_Position_*. Then, *N^AP^* is an *AP* cluster head neighboring set. During each iteration *h* of Algorithm 2, we first compute the virtual force of each cluster (Line 3) and then determine *d_in_* (Lines 5–10) and the new position of the cluster head (Lines 12–16). Line 18 involves moving the cluster head to a new position. Note that the positions are within the region *A*. When *h* achieves the max value, *Max_interation*, or $\left|{\vec{F}}^{h}|\;\geq |{\vec{F}}^{h-1}\right|$, the iteration is stopped, and we return $AP_{Position\_new}$.
**Algorithm 2:** The minimum inter-cluster interference strategy based on improved virtual force and motion.
**Input**: $AP=\{ap_{1},ap_{2},\cdots ,ap_{z}\}$ and *N*^AP^, $AP_{P_{osition\_old}}=\{\;ap_{Positon\_old}^{1},ap_{Positon\_old}^{2},\cdots \}$, *F_TH_* **Output**: *AP_Positon_new_*1Initialization: *Max_interation*; *C*; $ap_{Positon\_old}$2**While** (*h* < *Max_interation*)3 **if**
$|{\vec{F}}^{h}|\;\geq |{\vec{F}}^{h-1}|\;$4  **for**
*j* = 1; *j* ≤ |*AP*|; *j*++ do5   Calculate the virtual force $\vec{F_{j}}$ of *ap_j_* according to Equations (15)–(18);6   **if**
$\vec{F_{j}}$ > 07   Calculate the maximum move distance *d_p_* according to Equation (20);8    **if**
*d_p_* > *d_move_step_ //* Ensure that the cluster head covers its target points9     *d_in_* ← d*_move_step_*10    **else**11     *d_in_* ← d*_p_*12    **end if**13    Update the new position of $ap_{Positon\_old}$ according to Equation (22)14    **if**
*ap_position_new_* ∈ *A* // Evaluate whether the new position is in *A*15     *ap_position_new_* ← *ch_position_new_*16    **else**17     *ap_position_old_* ← the corresponding boundary value of *A*18    **end if**19   Move the cluster head to the new position20   *ap_position_new_* ← *ch_position_new_*21   **end if**22   Compute the total virtual force of the entire network $\vec{F}$23  **end for**24 **end if**25**end while**26**Return**
*AP_Position_new_* // Return the new position of the cluster heads

## 5. Simulation Results

To evaluate the deployment algorithm of cluster heads of the IMWN, the deployment simulation environment is built in the simulation software. The performance of the algorithm is tested numerically. By comparing the algorithm with similar current approaches, the performance of each algorithm is evaluated via the following three aspects: (1) the cluster head number (CHN), (2) the location changes of CHs using VFM, and (3) the inter-cluster interference target point number.

### 5.1. Experimental Environment

The simulation settings are described as follows. The simulation area *A* is represented as *A* = 1000 m × 1000 m. The discrete path points and static nodes constitute the set of target points *P*^′^ such that the discrete path points constitute 20% of the total. The transmission range *R* of every target point is 50–100 m, and the traffic load of each node is 10. The number of target points within the simulation area *A* is 50–100. Using the algorithm proposed here, the average results are obtained by repeating the simulation 100 times, comparing the algorithm to the regular hexagon deployment [36] (RHD), random placement (RP) [37], and K-means (KM) [38] methods. Additionally, the other parameters of the proposed method are given as follows in Table 1. In the RHD method, we use communication range *R* as the hexagon side length; in the KM method, we use the increasing K value to cover all target points. Similarly, in the RP method and in our proposed method, the cluster heads are deployed until the all wireless nodes and target points are fully covered.

### 5.2. Results Analysis

**Figure 3:**
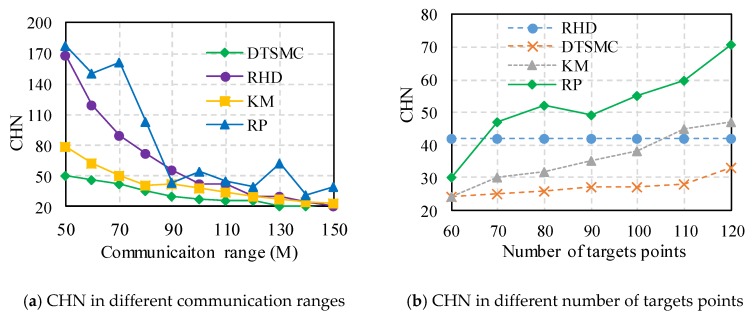
The results of CHN in different methods.

**Figure 4:**
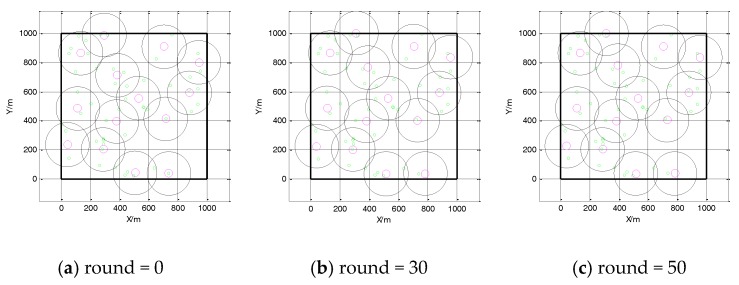
Location changing in different iteration with virtual force and motion.

We use the number of cluster heads required to fully cover the target node as the optimization objective to assess the algorithms. Figure 3 shows the network deployment effect diagram using the proposed DTSMC with 50 target points and 14 cluster heads. The results show that the target points (green points) are fully covered by the cluster heads (red circle) as show in Figure 4a. The results also demonstrate the efficiency of the proposal. By keeping the number of target points (*n* = 100) (and their locations) unchanged and by increasing the communication radius from 50 to 150 m, the CHN curves vary in different ways (DTSMC, KM, RP, and RHD), as shown in Figure 3a. Generally, the CHN decreases as the communication radius *R* increases. When the communication radius is high, the cluster heads cover more area and more wireless nodes and target points. The CHN of the RP achieves a maximum value, and the RHD increases in value. The DTSMC also obtains the minimum CHN. Thus, the DTSM can achieve the best performance relative to the other algorithms in terms of CHN. When the communication radius is 100 m, the DRSMC can save 35%, 28%, and 50% CHN relative to RHD, KM, and RP, respectively.

Similarly, Figure 3b shows the CHN figure with a communication radius *R* = 100 m and where the target points increase from 60 to 120. In general, the CHN for the remaining algorithms, excluding RHD, increases with the number of target points, and RHD remains unchanged. In the RHD (regular hexagon deployment) method, when the communication range is given, the number of cluster heads reaches a constant value. Therefore, when the number of target points increases, there are no changes to the CHN in RHD. Clearly, DTSMC requires that the smallest CHN be below several target points. When *n* is larger than 70, RP achieves the highest possible rising rate of CHN. If *n* is larger than 105, the CHN of KM is more than that of RPH. Whether the radius of communication increases or the number of target points increases, the proposed strategies can find the smallest CHN. In other words, our deployment strategy has an advantage in terms of the number of cluster heads and the performance with full coverage. The reasons are that in our proposal, by using a distribution Tabu search, it is easy to obtain optimal results.

The subfigures of Figure 4 show the changing snapshot locations of cluster heads such that *R* = 150 m and *n* = 50. The red circles are the centers of the cluster heads, the green circles are the target points, and the black circles are the cluster communication radii and the cluster ranges. Figure 4a shows the initial configuration, Figure 4b shows the configuration after 50 rounds, and Figure 4c shows the final deployment. At first, several inter-cluster interference target points exist. When the round is 23, the location of cluster heads will not change; if the round is more than 50, the number of inter-cluster interference target points decreases. It is shown that our proposal is convergent, and the cluster head deployment can enable full coverage for all target points. A comparison of Figure 4b with Figure 4c indicates that no difference exists in the location and deployment of cluster heads. Furthermore, the virtual force of the whole network achieves the minimum value, and the inter-cluster interference is the smallest final configuration. Moreover, Figure 4 shows the feasibility and convergence of the algorithms.

**Figure 5:**
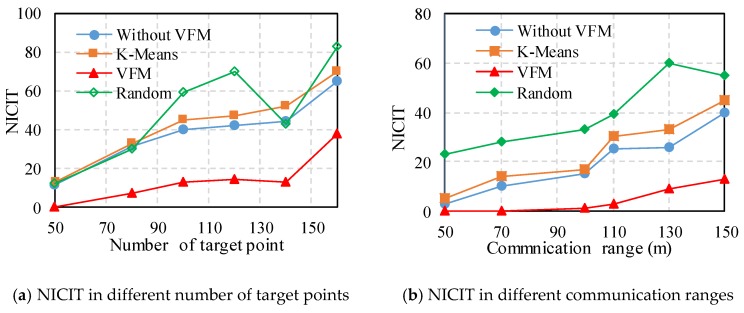
The results of NICIT in different methods.

We use the number of inter-cluster interference targets (NICIT) to assess the reliability of the deployment and efficacy of the VFM. Figure 5a shows the NICIT curve resulting from four strategies (without VFM, KM, or RP) with *n* targets (such that *n* = 100) and with various communication radii from 50 to 100 m. The NICIT increases after the targets are added, and RP yields the worst effects. VFM provides the best performance, particularly if *R* is less than 140. When *R* is more than 90 m, the NICIT greatly increases for the remaining methods, except for VFM. The NICIT for *R* = 150 m, with different methods, is the number of target points from 50 to 150, as shown in Figure 5b. Generally, all NICIT values rise when *n* is added, as a result of more target points increasing the probability of inter-clutter interference. Similarly, VFM produces the best performance in terms of NICIT. Relative to KM, without VFM and RP, VFM reduces the NICIT values by 93%, 94%, and 94%, respectively. Moreover, our proposal can decrease the inter-cluster interference and increase the reliability of the IMWNs. Since our algorithm adopts the virtual force and motion on cluster heads to reduce inter-cluster interference, the presented strategy always obtains the lowest NICIT compared with other methods.

**Figure 6:**
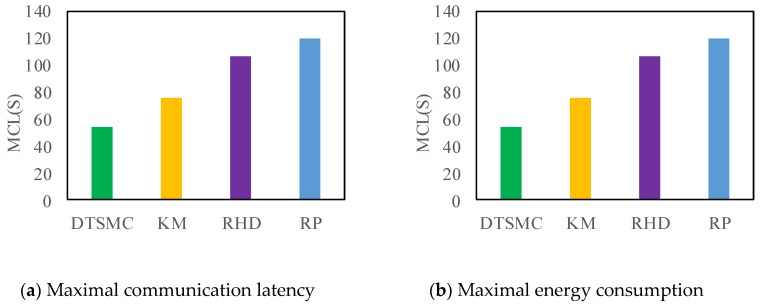
The maximal communication latency (MCL) and maximal energy consumption (MEC) in different methods.

We use the MCL and MEC as evaluation criteria in one cycle for network communication latency and energy consumption. Figure 6 shows the MCL using the proposed DTSMC and other methods with 50 target points for full coverage. Figure 6a shows the results of MCL of all methods. The results demonstrate that the DTSMC has the lowest MCL. Namely, when *n* = 50 and *R* = 100 m, the DTSMC has low max communication latency and will perform better in real time. DRSMC can save MCL more than 5%, 10%, and 20% relative to RHD, KM, and RP, respectively. In the same way, Figure 6b demonstrates the MEC of the different strategies. The DTSMC shows the same trend; our proposal achieves the lowest MEC. The DTSMC, compared with other algorithms, consumes less energy. In sum, employing the maximal communication load limit during the cluster head deployment, DTSMC performs better in terms of MCL and MEC. 

## 6. Conclusions

IMWNs form a significant basis for connecting a smart factory, an Industry 4.0 site, or other intelligent manufacturing systems. In clustered networks, full coverage and the reliable deployment of cluster heads are directly related to the performance of the entire system, such as real-time response and reliability, making such factors a topic of active research in academia and industry. Wireless node deployment is a hot topic for wireless networks, as previous work has not considered the properties of industrial applications, and the current approaches are not suitable for manufacturing systems. We employ an appropriate system model to simplify the static and mobile node coverage to target coverage. We divide the full target coverage into two stages: maximal cliques for full coverage and minimal inter-cluster interference. Then, we apply the optimal strategies by adopting a distributed double-layer Tabu search and improve the virtual force and motion. We developed a simulation of our proposal, and the results demonstrate that our strategies can reduce the number cluster heads and increase reliability by comparison to other traditional methods such as RHP, KM, and RP. Meanwhile, comparing results of the maximal communication latency and maximal energy consumption of IMWNs demonstrates that our methods can reduce energy consumption and increase real-time communication. In the future, we will study the changing communication range and loads. We also plan to apply the proposal to our prototype platform and other industrial applications. 

## Figures and Tables

**Table 1:** Parameters values used in the simulation.

Parameter	Value	Description
*R*	50, 100, 150, 200	Communication radius
*n*	50, 60, 70, 80, 90, 100	Number of target points
*F*_TH_	0	Virtual force threshold
*D_i_*	100 Mb	Communication load of wireless node
*D*max	500 Mb	Maximum allowable communication load
ς	0.1 J/Mb	Coefficient of communication energy consumption
ξ	0.08 J/Mb	Coefficient of wireless channel energy consumption
*Prob*	20%	Data recommunication probability
*Vc*	10 Mb/s	Communication rate
D*_move_step_*	10, 20, 30	Moving step length
*Max_iterations*	100	Number of iterations
